# First person – Francesca Mastropasqua and Marika Oksanen

**DOI:** 10.1242/bio.060170

**Published:** 2023-10-17

## Abstract

First Person is a series of interviews with the first authors of a selection of papers published in Biology Open, helping researchers promote themselves alongside their papers. Francesca Mastropasqua and Marika Oksanen are co-first authors on ‘ Deficiency of the Heterogeneous Nuclear Ribonucleoprotein U locus leads to delayed hindbrain neurogenesis’, published in BiO. Francesca is a Senior research specialist in the lab of Kristiina Tammimies at the Karolinska Institutet in Sweden, investigating the molecular effects of genetic mutations. Marika is a PhD student in the same lab investigating RNA-binding proteins and their ability to control gene expression.


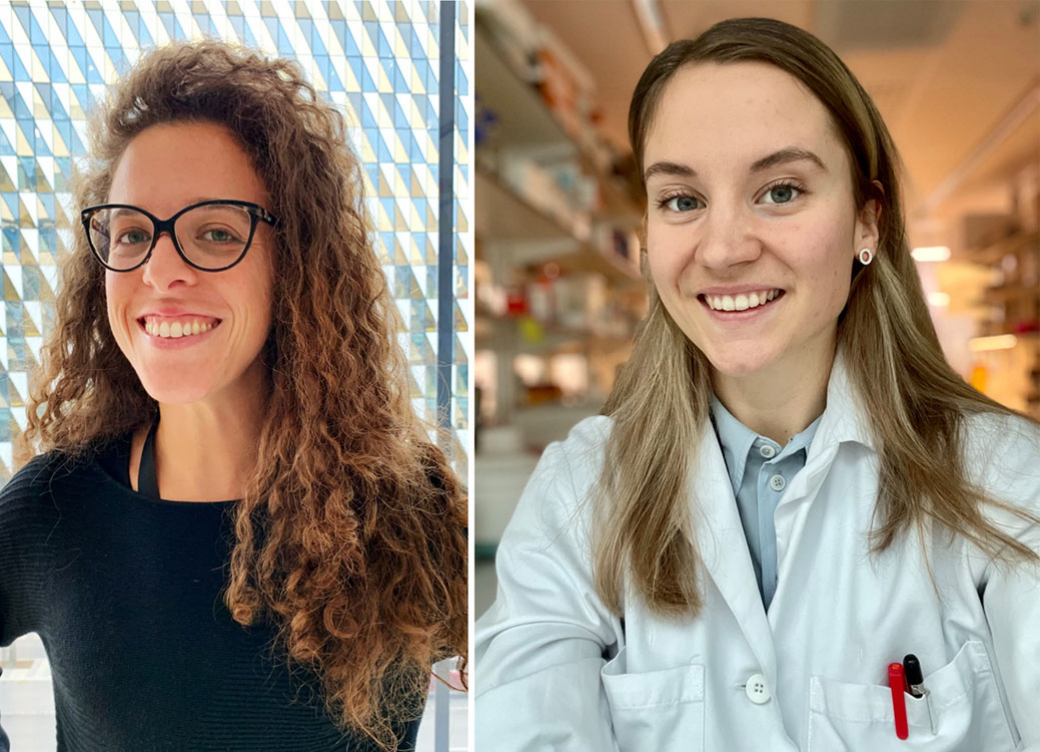


**Francesca Mastropasqua and Marika Oksanen**

**Describe your scientific journey and your current research focus**

We both are molecular biologists by training who lately have being focusing on neuronal development and neurodevelopmental disorders, in line with the interests of our current research group. The group is mainly working with understanding the genetic and molecular architecture in neurodevelopmental disorders and how genetic variations influence severity and intervention outcomes in affected individuals. In this frame, we work on bioinformatic analyses of omics data as well as on ad hoc cellular and molecular assays to validate the findings.

**Who or what inspired you to become a scientist?**

**F.M.:** My mother used to teach molecular biology at the University, and I grew up witnessing the ‘second family’ that she put together in her lab, how much ‘fun’ science could be and how good it was to find solutions and answers to big questions together. This inspired me to become a scientist and to always work towards a similar level of collaboration and closeness with my colleagues and collaborators that I witnessed in my youth.

**M.O.:** During high school, I was on a biology course focusing on biotechnology, which inspired me to apply to a university program with a similar focus. I majored in molecular biology and minored in economics, initially planning to transition into the field of marketing within life science industry. A single internship in a research lab was all it took to open my eyes for the vast possibilities in academic research and I never looked back!

**How would you explain the main finding of your paper?**

Some cases of individuals with neurodevelopmental disorders (e.g. autism, intellectual disability) have been associated with mutations in specific genes. Nonetheless the molecular effects of these mutations on the physiology of the cells have not always been explained. In this paper we show that the mutations affecting the *HNRNPU* gene induce changes in the organization of the DNA inside the nuclei of the cells and in the expression of hundreds of other genes known to be involved in cellular proliferation and neurodevelopment. Our main finding focuses on the effects of the mutations at the cellular level, where they cause the impairment of the physiological switch from dividing undifferentiated cells to neuroprogenitors, delaying the overall differentiation process.

**Figure:**
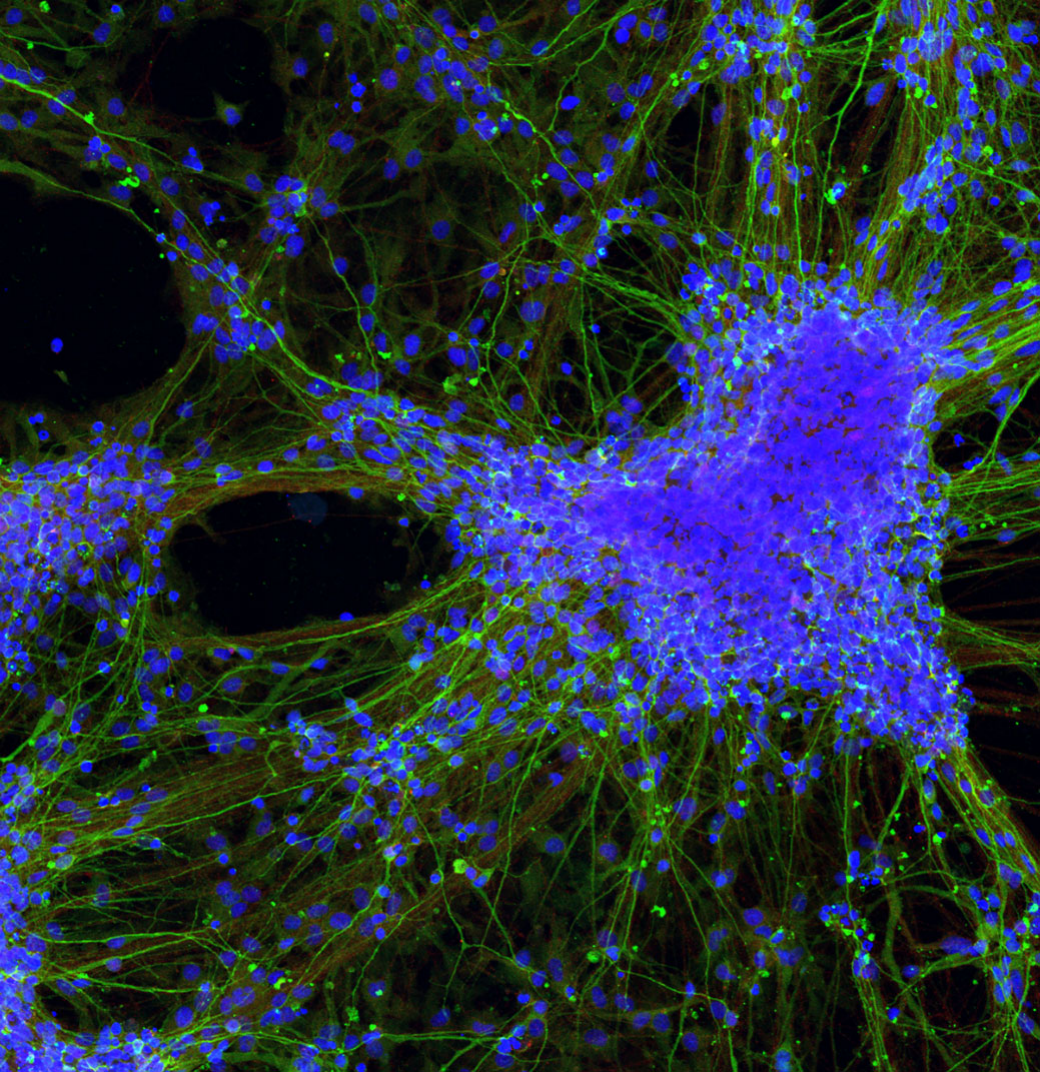
**Neurons ‘in love’.** Immunofluorescent picture of neuronal cells after 28 days of differentiation from neural stem cells. Green: Tuj-1; Blue: Hoechst.

**What are the potential implications of this finding for your field of research?**

Our study shows that the effects of the mutations in neurodevelopmental disorders need to be studied in very early developmental models, since the first effects that generate the following cascade might be very early on during the development. We, as well, show that the complexity in understanding the genetic of these kinds of disorders can reside in the multiplicity of roles that the proteins expressed by these genes can exert within the cells, suggesting that the integration of multiple kind of analysis are beneficial in order to obtain a broader picture of the biology of cells carrying the mutations of interest.

Our study shows that the effects of the mutations in neurodevelopmental disorders need to be studied in very early developmental models

**What piece of advice would you give to the next generation of researchers?**

Science research can be extremely demanding. Our best advice is to make sure you choose research topics and projects that genuinely interest and excite you. At the same time, aim to create a group environment that supports you and where colleagues are friends and family. Passion for your work and the people who share it with you will sustain you through the inevitable challenges and setbacks in the research process and make sure you can enjoy your research.

Passion for your work and the people who share it with you will sustain you through the inevitable challenges and setbacks in the research process and make sure you can enjoy your research.

**What's next for you?**

We are currently working on a follow-up study on HNRNPU in neuronal development, and Marika hopes to finish her PhD in this subject within the following year.
